# Hardware-in-the-Loop Simulations with Umbra Conditions for Spacecraft Rendezvous with PMD Visual Sensors

**DOI:** 10.3390/s21041455

**Published:** 2021-02-19

**Authors:** Ksenia Klionovska, Matthias Burri

**Affiliations:** German Space Operations Center, German Aerospace Center, Münchener Straße 20, 82234 Weßling, Germany; matthias.burri@dlr.de

**Keywords:** PMD sensor, close range rendezvous, hardware-in-the-loop simulations, illumination conditions

## Abstract

This paper addresses the validation of a robust vision-based pose estimation technique using a Photonic Mixer Device (PMD) sensor as a single visual sensor in the close-range phase of spacecraft rendezvous. First, it was necessary to integrate the developed hybrid navigation technique for the PMD sensor into the hardware-in-the-loop (HIL) rendezvous system developed by the German Aerospace Center (DLR). Thereafter, HIL tests were conducted using the European Proximity Operation Simulator (EPOS) with sun simulation and in total darkness. For the future missions with an active sensor, e.g., a PMD camera, it could be useful to use only its own illumination during the rendezvous phase in penumbra or umbra, instead of additional flash light. In some tests, the rotational rate of the target object was also tuned. Unlike the rendezvous tests in other works, here we present for the first time closed-loop approaches with only depth and amplitude images of a PMD sensor. For the rendezvous tests in the EPOS laboratory, the Argos3D camera was used at the range of 8 to 5.5 m; the performance showed promising results.

## 1. Introduction

Autonomous space rendezvous is an important part of On-Orbit Servicing (OOS) and Active Debris Removal (ADR) missions. The demands for these missions are increasing continuously due to the high number of non-operational satellites, spent rocket stages and other different pieces of debris [1], which threaten the International Space Station and other operational satellites. During OOS and ADR missions, different services can be provided: replacement of failed subsystems, refueling of propellant, replenishment of a spacecraft’s components (e.g., batteries or solar arrays), extension of a mission (e.g., software and hardware upgrades) or complete deorbiting of a non-operational space object. OOS and ADR mission scenarios consider at least two space objects: a servicer satellite and a target object. In order to accomplish aforementioned tasks, the servicer satellite has to approach the target at close-range. When the target is non-cooperative, there is no information about its position and orientation; any patterns and visual markers for the visual navigation are absent. The target object may tumble, making it more difficult to determine its pose.

Different visual sensors have been tested for rendezvous scenarios. Strengths and weaknesses of these sensor are compared in the literature. Monocular cameras require an external source of illumination, but are small in size and have low power consumption. A full pose estimate is possible because, for rendezvous in space, the scale of the approached target is usually known or can be estimated. Estimations were used for visual navigation in relation to non-cooperative targets by Gaias et al. [2], Sharma et al. [3] and Bennighoff et al. [4]. Lingenauber et al. [5] presented a plenoptic camera for autonomous robot vision during OOS missions at very close range (as close as 2 m from a satellite mockup). The use of stereo vision allows the tracking [6,7] and the identification [8] of an illuminated non-cooperative target. Yilmaz et al. considered infrared sensors for relative navigation for future ADR missions [9]. The sensitivity to the radiation emitted by the target allows operation in darkness, and pose estimation precision is limited by the sensor’s resolution [10]. Active scanning light detection and ranging (LIDAR) sensors have already been tested for autonomous rendezvous in real space missions [11] and on the ground [12,13]. Operations in darkness are possible, but their size and the moving parts make them expensive and fragile.

The use of time-of-flight sensors has been presented in the work of Ventura [14]. The use of active visual sensors with the Photonic Mixer Device (PMD) technology for the close rendezvous phase is presented in the works of Tzschichholz [15] and Klionovska et al. [16]. Due to the fact that PMD sensors are built using CMOS fabrication technology, they had attractive prices years ago, before low cost automotive LIDARs came onto the market. PMD sensor technology has never been used in any real space application before. The lack of moving parts makes it mechanically robust, and as an active sensor it has the potential to operate in complete darkness. This fact raised an interest in testing it on ground more thoroughly in a closed-loop rendezvous simulation, in order to evaluate the technology for potential use for future missions.

For a rendezvous with a non-cooperative target, the choice of an appropriate pose estimation technique is relevant to converting raw sensor data into information usable for guidance, navigation, and control systems. Random Sample Consensus (RANSAC) is the state-of-the-art iterative parameter estimation technique for data with outliers, and it is used for pose estimation with 3D point clouds from LIDARs and stereo vision systems [13,17]. Some simple deterministic methods such as Principal Component Analyses (PCA) and Singular Value Decomposition (SVD) have been used to find the orientation of the main axis of the target in proximity operations in [15,18]. The Iterative Closest Point (ICP) [19,20] algorithm with its different modifications is one of most popular algorithms for pose estimation with 3D point clouds. Feature-based 2D pose estimation techniques with detection of contours and edges of objects in space are presented in works of Cropp [21], D’Amico [22] and Petit et al. [23]. There are also optical flow methods [24] that consider pixel intensities in the consecutive images, and template-based techniques [25,26]. A recent trend is the research with Convolutional Neural Network (CNN)-based algorithms for the 6D pose estimation of non-cooperative targets using 2D vision systems [27,28].

Without a robust Guidance Navigation and Control system (GNC) [29], an autonomous rendezvous cannot be achieved. Currently, an advanced GNC system is being developed at DLR within the Rendezvous, Inspection, Capture, Detumbling for Orbital Servicing (RICADOS) project [4,30,31]. The hardware-in-the-loop (HIL) simulation allows one to test approach trajectories, visual optical sensors and image processing algorithms in real time on the ground with different illumination conditions. The European Proximity Operations Simulator (EPOS) at DLR is used as a HIL simulator for the final rendezvous phase (starting from 20 m). The Argos3D camera with a PMD sensor is integrated in the EPOS facility. The developed navigation algorithm for the PMD sensor is part of the current GNC system.

The main subject paper is the evaluation of navigation performance in closed-loop rendezvous approaches using the PMD sensor as the single visual sensor. Specifically, we evaluate the effects of illumination conditions and the influences of the rotational rate of the target on the accuracy and stability of the navigation system. The pose estimation and navigation techniques developed for the PMD sensor have been described in previous work [16,32]. Our previous work [32] used various amounts of recorded images for the rendezvous simulations. Thus, the output of the navigation system was not fed into the control system. In this paper the processed PMD sensor measurements are used for the real-time control of the approach trajectory. It is a big step forward towards a fully autonomous approach with PMD measurements.

This paper is organized as follows. Section 2 describes the HIL rendezvous system, the PMD sensor and the applied pose estimation algorithms. We approached a rotating target in total darkness with strong side illumination. We also compared approaches with different rotational speeds along the principal axis. Section 3 presents the results and discussion of the closed-loop rendezvous scenarios, and the conclusions are in Section 4.

## 2. Materials and Methods

In this section we present the HIL rendezvous system, the characteristics of the PMD sensor in question, short descriptions of the navigation algorithms and the simulation scenario.

### 2.1. Hardware-in-the-Loop Rendezvous System

**Figure 1:**
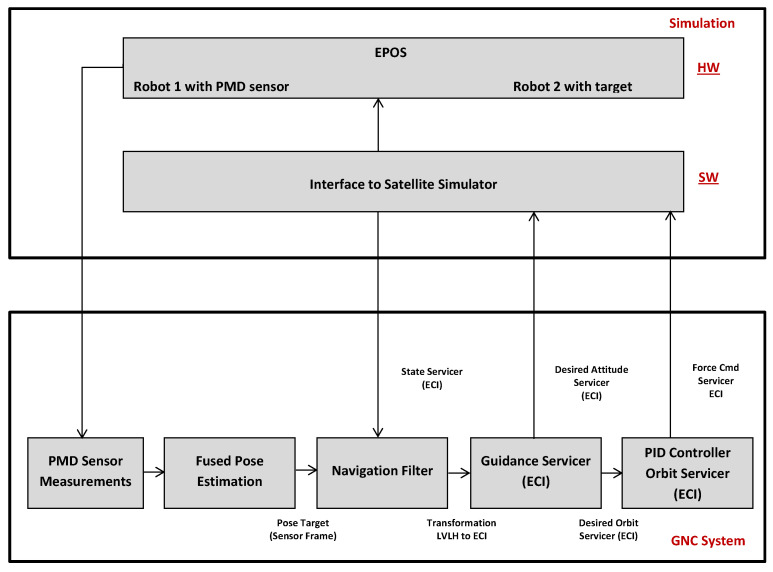
Illustration of the hardware-in-the-loop rendezvous system.

**Figure 2:**
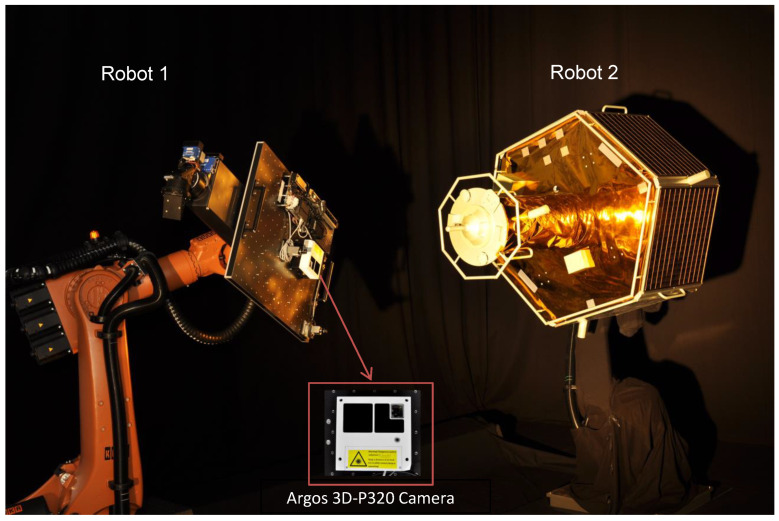
EPOS facility: on the left, a robot carries a Photonic Mixer Device (PMD) sensor and the one on the right has a mounted mockup of a satellite.

The complex HIL rendezvous system used for the experiments consisted of the simulation part and the GNC system; see Figure 1. The simulation part consisted of a software-based satellite simulator and the robotic HIL test facility EPOS [33,34] presented in Figure 2. The advanced GNC system included measurements from the PMD sensor, pose estimation algorithms, a navigation filter and guidance and control functions.

As shown in Figure 2, the EPOS rendezvous simulator consists of two robots: Robot 1 is able to move along a rail system and robot 2 is fixed at the end of the rail system. The mockup of a satellite (target) is mounted on the fixed robot, whereas the other robot (servicer) carries a Argos3D camera with the PMD sensor inside of a white housing.

Let us describe step-by-step the flow of the diagram in Figure 1. PMD sensor images of the target object are acquired. At the stage “Fused Pose Estimation”, those images are processed to estimate the position and orientation of the non-cooperative target. The blocks “PMD Sensor Measurements” and “Fused Pose Estimation” can freely be substituted with other visual rendezvous sensors and pose estimation techniques. Nevertheless, they are kept constant for the described setup. The noisy measurements are passed through the navigation filter described in [35], and we get a pose with minimized noise in the Earth Central Inertial (ECI) system. Following the data flow of Figure 1, the guidance system computes the desired servicer attitude with the output from the navigation filter in block “Guidance Servicer (ECI)”. A Proportional Integral Derivative (PID) controller in the block “PID Controller Orbit Servicer (ECI)” translates this result to the control forces needed to keep the servicer on the desired trajectory. The satellite simulator, at the stage “Simulation”, computes the dynamic motion of the servicer and target, and then forwards this data to EPOS. The EPOS robots move relative to one another according to a real-time attitude and orbit dynamics simulation, just as a servicer and target would move in orbit.

### 2.2. Argos 3D-P320 Camera

The Bluetechnix (current BECOM) Argos 3D-P320 camera (white camera in Figure 2) contains the PMD sensor. The PMD sensor is a ranging device that provides a depth image for every frame. The depth measurement of every pixel is obtained considering the phase shift between the emitted signal of LEDs and the signal reflected from the target. The camera in the current setup has 12 LEDs. For detailed descriptions of the PMD’s operational principle and depth calculation per pixel, please refer to the works of Langmann [36]. The technical characteristics of the current PMD sensor are presented in Table 1.

As shown in Table 1, the resolution of the current PMD sensor inside the Argos 3D-P320 camera is relatively small compared with the traditional CMOS image sensors available on the market. Both sensor families use integrated circuits placed inside each pixel to convert the incoming light into a digital signal. For the depth calculation, a certain amount of electronics is required, resulting in larger pixels. The current generation of PMD sensors can only achieve pixels of ca. 10 microns, whereas the CMOS camera can reach a pixel size of 1 micron [37].

**Figure 3:**
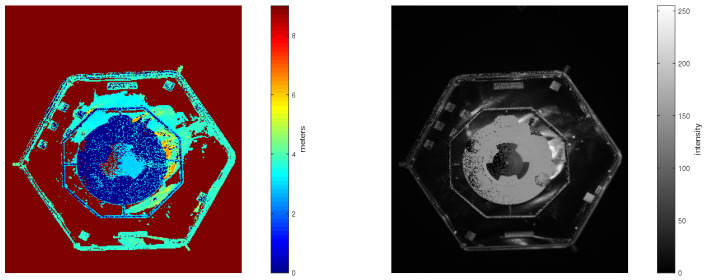
(**Left**): Depth image recorded with an additional illumination spot. The colorbar represents distance measured to the object in meters. (**Right**): Amplitude image recorded with an additional illumination spot. The colorbar represents intensity in the range from 0 to 225.

On top of depth information, the PMD camera provides a co-registered amplitude gray-scaled image. The amplitude image reflects the strength of the signal returned by the target. Single examples of depth and amplitude images taken in the EPOS laboratory with strong side illumination are presented in Figure 3.

**Figure 4:**
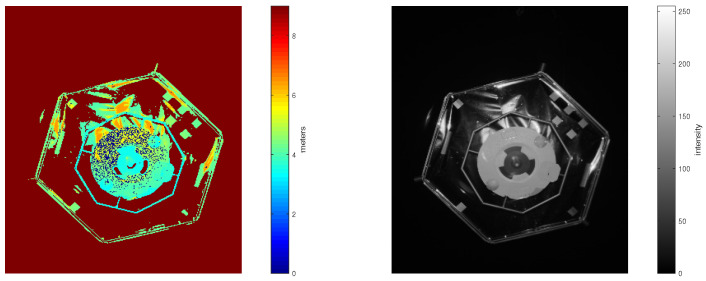
(**Left**): Depth image recorded in complete darkness. The colorbar represents distance measured to the object in meters. (**Right**): Amplitude image recorded in complete darkness. The colorbar represents intensity in the range from 0 to 225.

The PMD sensor is an active sensor that can operate even in complete darkness without any flash light. This is a big advantage for a future mission planning. A depth and an amplitude image in “umbra conditions” in the EPOS laboratory are shown in Figure 4.

When comparing these two pairs of depth and amplitude images with images taken in the presence of illumination in Figure 3 and images taken in total darkness in Figure 4, one can hardly notice any differences. Even so, the images in Figure 3 are a bit more noisy than in Figure 4. This is due to different systematic and non-systematic errors in PMD sensor measurements [38]. There are several methods for error compensation, which are out of scope in this paper.

An accurately calibrated visual camera is a prerequisite for image processing. Like usual mono- or stereo cameras, the PMD sensor needs to be calibrated. For this work we considered the camera calibration process as an estimation of the camera model (intrinsic calibration) and estimations of the position and orientation of the PMD sensor frame in the camera housing (hand-eye calibration) with respect to breadboard of robot 1 of Figure 2. The DLR CalDe and DLR CalLab calibration toolbox [39] has been used during the calibration procedure. The step-by-step calibration process of the current Argos 3D-P320 camera is described in this work [40].

### 2.3. Fused Pose Estimation

**Figure 5:**
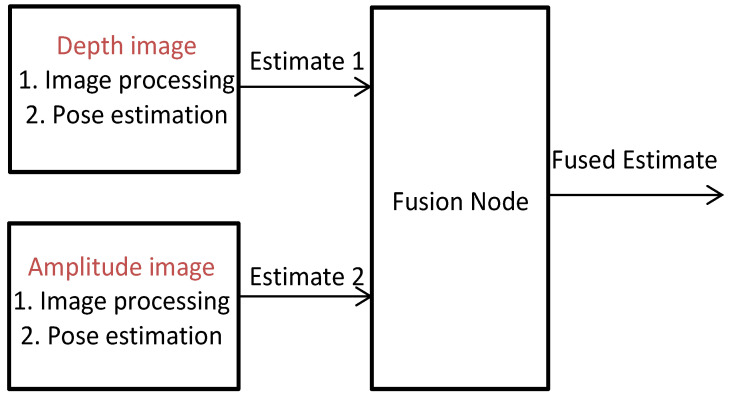
Fused pose estimation.

The fused pose estimation in the rendezvous system includes the pose estimation algorithms for the target object. The concept of the fused pose estimation technique arose after some tests with the current PMD sensor in the EPOS laboratory. Here the term fused pose estimation means the following. Instead of using only one pose estimation technique for the depth image of the PMD sensor, a second independent pose estimation method for the amplitude image is applied; see Figure 5. As a result, there are two estimated vectors of position and orientation, which are fused together for an unique pose.

In Klionovska et al. [16], we presented for the first time the possibility of using the amplitude image of the PMD sensor for pose estimation. The quality of the 2D gray-scaled amplitude image is sufficient to provide image processing and apply a stable pose estimation technique. From pose estimation techniques for 2D vision presented in Section 1, we chose an image processing method with low-level feature detection using the Hough line transform. Based on this transform, the straight lines forming the frontal hexagon of the satellite mockup and the endpoints of these lines are detected. The pose estimation consists of the least square minimization problem for the matches between the detected 2D points and the known 3D points of the target model. The minimization is implemented with a Gauss–Newton solver. The pose estimation technique for the depth image is based on the modified version of state-of-the-art Iterative Closest Point (ICP) algorithm, which uses the reverse calibration method for the neighbor search. In Klionovska et al. [32], the following tendency has been experimentally shown. The distance component is more accurately estimated from a depth image, and the amplitude image leads to a more precise attitude estimate. In order to get a final pose state that outperforms two separated local measurements, the weighted average [41] algorithm is applied for the measurement fusion. For a detailed description of each method, please refer to [42].

The great advantage of the proposed architecture is that no additional measurement sensors are required. Additionally, with the distributed architecture the measurements from both channels of the sensor do not need to be aligned, as they are in the same coordinate system.

## 3. Results and Discussion

### 3.1. Spacecraft Rendezvous Scenario

For the simulation scenarios we chose the nearly circular orbit of the ENVISAT satellite, which can be considered as a potential candidate for an ADR mission. The parameters of the orbit are the following: a perigee of 771.7 km, an apogee of 773.5 km, a semi-major axis of 7143 km, an inclination of $98.2^{\circ }$ and an orbit period of about 100 min. The PMD sensor will be active during the final phase of the rendezvous approach.

Using the rendezvous console of the HIL rendezvous system, the straight line approach guidance mode is activated by setting the start (8 m) and end (5.5 m) points. The distance corresponds to the distance between the centers of mass of the two spacecraft. This limited range span was chosen based on the following factors. The optical power of the illumination kit integrated in the PMD sensor restricts the maximum starting point of the rendezvous. The minimum distance limit results from the combination of the size of the existing mockup, the sensor’s field of view and the pose estimation algorithms that need to see the full target. Some points of the detected front hexagon are outside the image when the servicer with the PMD sensor comes closer than 5 m.

Four test scenarios have been simulated on EPOS; see Table 2. Test I and test II represent approaches with sunlight; the servicer approached the target with illumination from the side. In test III and test IV, the rendezvous tests took place in total darkness to simulate an approach in umbra conditions. The approach velocity of the servicer in all rendezvous scenarios was 0.01 m/s. This velocity has proved to be safe for autonomous rendezvous with non-cooperative objects. In test I and test III the target rotated at 3 deg/s; in test II and test IV, 1 deg/s. These spinning rates were chosen relative to reliably observed rotational rates of ENVISAT satellite in 2012 and 2016 [43]. That made our test scenarios more realistic. Each test case was repeated and recorded five times.

We do not consider in this paper a situation where the target object rotates around another axis. However, it should be possible to track a space object by adjusting the fused pose estimation technique.

### 3.2. Numerical Results

**Figure 6:**
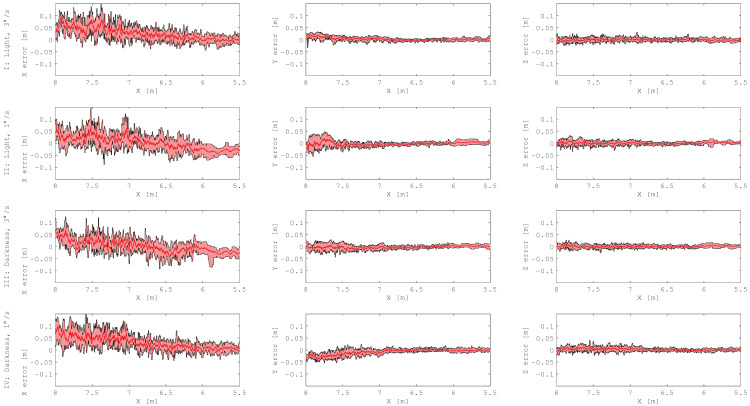
Position errors during approaches. The moving average (dark red line) is plotted over an error band limited by the moving minimum and maximum (black lines). For each test case, data from five repetitions were sorted by distance and then filtered using a 15-point filter window.

The results were processed in the servicer’s coordinate system. The x axis points towards the target. In Figure 6 the position errors of all five approaches in every test case are presented. For these approach trajectories, the errors for estimation of the distance were quite similar. In most cases the distance was slightly over that estimated; the maximum error was 16 cm. We observed that with a decreasing distance to the target, the estimated error dropped and did not exceed 5 cm at the point nearest to the target. The maximum position error for y and z axes in all test cases was 4.8 cm, but in general it was smaller to the error for the x axis. If we compare the approaches in total darkness to the approaches with an illuminated target, we see nearly identical errors. However, approaches in total darkness showed a less severe systematic offset. This was expected, since the PMD sensor was not affected by any illumination or reflected light.

**Figure 7:**
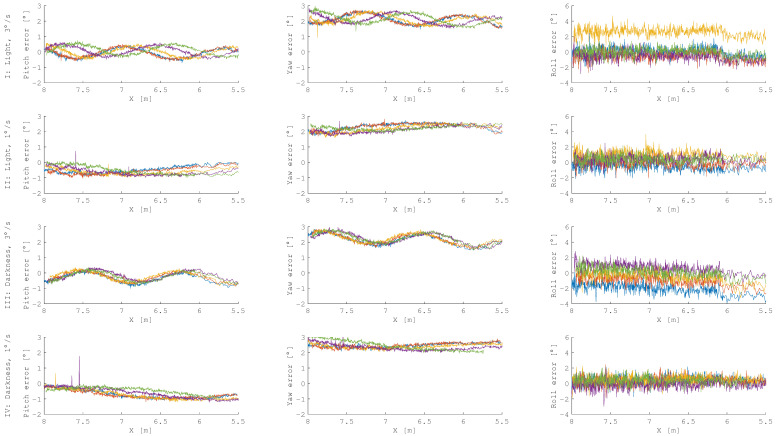
Attitude errors during approaches. There were five approaches for four test scenarios, and each approach is colored differently in the plots.

**Figure 8:**
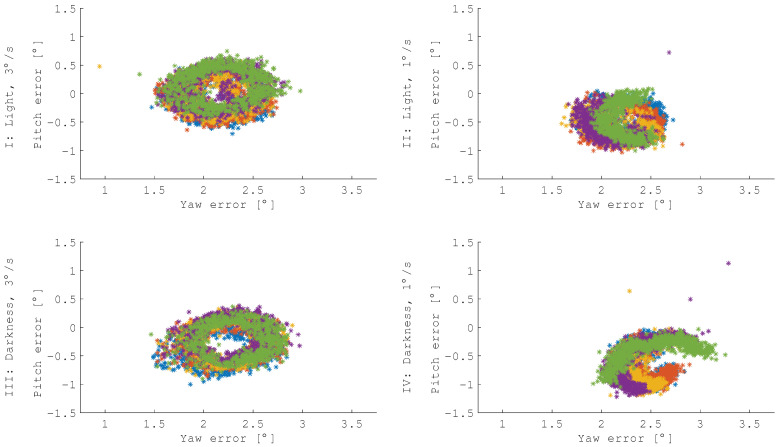
Orientation error around pitch and roll axes. The misalignment between the approach axis and the rotation axis of the target produces a circular shape, and the variance of the measurements makes the error plots have a donut shape. Every subplot has five donuts with different colors: approach 1—blue; approach 2—red; approach 3—yellow; approach 4—violet; approach 5—green.

Figure 7 shows the attitude errors. Looking at the plots of the roll angle, it is not difficult to notice that in test I and test III there were some approaches wherein errors were higher than in test II and test IV. The angular velocity of a target mockup affects the accuracy of the estimated roll angle. All deviations of pitch and yaw angle have a systematic sinusoidal error. The error frequency corresponds to the rotation frequency of the target, and the peak to peak amplitude is between 0.36 and 0.88 degrees. In Figure 8, the pitch error is plotted over the yaw error. There is a systematic offset in the yaw angle larger than 2 degrees. The amplitude and frequency of the systematic errors are similar for both axis. As a result, the plots for the faster rotating target show circles, whereas the slower rotating target did not complete a full revolution, and therefore, only arc-circles are visible. A detailed characterization of yaw and pitch errors for a sinus model with offset can be found in Table A1 in the Appendix A.

In Figure 6 and Figure 7, the density of the results in a range starting from 6 m evidently drops. This effect is related to the computational time of the pose estimation algorithm. The closer the servicer satellite is to the target, the more points there are in the recorded data, resulting in decreasing pose estimation frequency.

**Figure 9:**
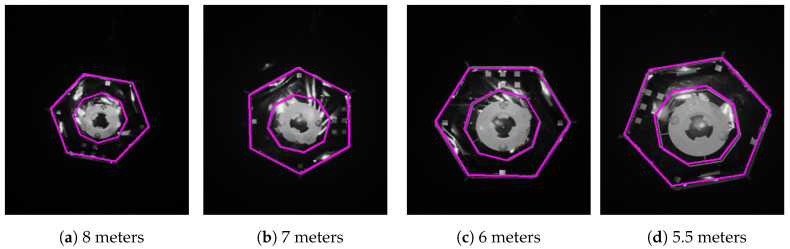
Projection of the estimated pose at different distances from the target satellite during the 5th approach of test IV in darkness. The target rotated at 1 deg/s.

**Figure 10:**
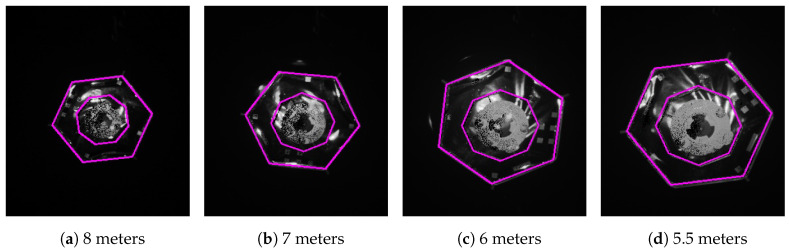
Projection of the estimated pose at different distances from the target satellite during the 5th approach of test II with additional illumination. The target rotated at 1 deg/s.

For visual representation of the results, we plotted some images with the estimated pose for the 5th approach of test II and test IV. Figure 9 and Figure 10 show four images of the mockup at different distances (from left to right): 8, 7, 6 and 5.5 m. The magenta contour in every image is a matched model with the estimated pose at that moment. We can observe a small misalignment of the projected model and the mockup in Figure 9d because of the 2 degree error.

When comparing the approach under umbra conditions visualized in Figure 6 with the approach with additional light in Figure 7, we can see that there are only a few additional reflections visible on the larger hexagonal surface of the target in the background. The amplitude of the points on the circular surface inside the octagon in the center of the target looks nearly uniform in umbra conditions. In the case with additional illumination, this surfaces looks much more noisy. This observation agrees well with the observation from Figure 6 that the approaches in darkness have less severe systematic offsets.

**Figure 11:**
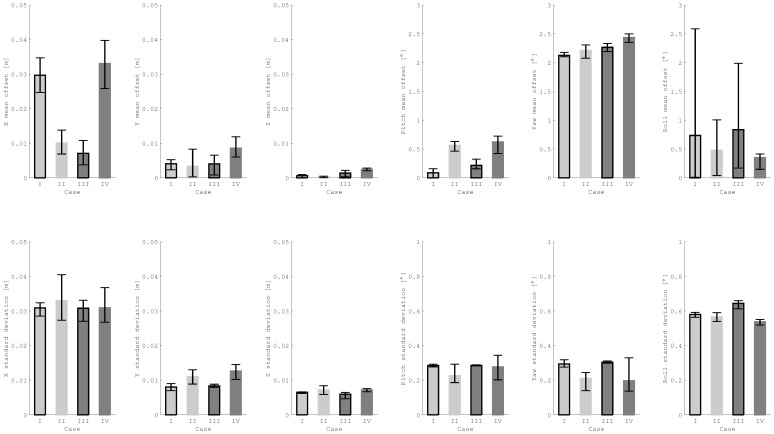
In the first row, the mean offset of PMD pose estimation is plotted as a rectangle. A line indicates the range between minimal and maximal offsets over five repeated approaches to the target satellite. In the second row the standard deviations of PMD pose estimation errors are plotted. The approaches with illumination are filled with light gray (test cases I and II), while the darker bars highlight the approaches in umbra conditions (test cases III and IV). The approaches towards a target rotating at 3 deg/s are marked with a black edge (test cases I and III) and the box without edges stands for a target rotating at 1 deg/s (test cases II and IV).

Figure 11 summarizes the mean offsets and standard deviations for rotation and translation errors for all approaches within four tests. The maximum mean offset for the estimated distance appeared in test IV—4 cm, whereas the minimum was within test III—0.5 cm. The standard deviation for the distance seems to have been around 3 cm for most of the approaches; for a few outliers it was up to 4.1 cm. The position errors of the y and z components also appeared to be around 1 cm or even smaller. Concerning the attitude errors for both simulated rotational speeds of the target, the standard deviations of three angles were below 1 degree. The mean offsets for the roll angles fluctuated far more over the repetitions, especially when the target rotated at 3 deg/s. A possible explanation could be that the the roll angle was computed using the position information of the corner pixels of the sensor, whereas the other two angles were mostly computed from differences of distances.

## 4. Conclusions

An HIL rendezvous simulation with a single PMD sensor was presented in this paper. A set of test rendezvous scenarios were designed and executed in order to compare the stability and accuracy of visual navigation. The pose estimation techniques used for the visual navigation were briefly described in the Materials and Methods section. In that section the PMD sensor and its characteristics were also introduced. The highlight of the paper is the presentation of different closed-loop tests with a single PMD sensor at EPOS laboratory. We tested rendezvous scenarios with an additional spotlight and in complete darkness. There were also tests with different rotational rates for the target.

In the Results and Discussion section, we compared the pose estimation errors during the tracking for the test cases when the target rotated with speeds of 1 and 3 deg/s. For all cases, the fused pose estimation technique was able to estimate the position and orientation of the target during the whole tracking phase. The increased rotational rate of the target did not cause significant errors in the estimated roll angle. Within the approaches of test III and test IV, we showed the possibility of navigating to the target object at close-range with a current PMD sensor and without any additional flashing light. In general, throughout all approaches, the tracking was stable, without any interruptions or breaking.

Some further improvements are suggested: Minimization of the PMD sensor’s errors, which affect the final estimated pose of the target. In order to support approaches that come closer to the target, the pose estimation algorithm can be improved to work with only visible parts of the target. Replacing the LED illumination unit with laser diodes is another option for the extension of the operational range of the current PMD sensor.

## Figures and Tables

**Table 1:** Technical data of the PMD sensor inside of the Argos 3D-P320 camera.

Parameter	Numerical Values
Field of View	28.91 × 23.45 deg
Resolution of the chip	352 × 287 pixels
Integration time	24 ms
Frames per second	45
Modulated frequencies	5.004 MHz, 7.5 MHz, 10.007 MHz,
	15 MHz, 20.013 MHz, 25.016 MHz, 30 MHz
Mass	2 kg
Power Consumption	<25.5 W

**Table 2:** Overview of the test cases.

Case	Illumination	Target Rotation
Test I	Target enlighted	3 deg/s
Test II	Target enlighted	1 deg/s
Test III	Umbra conditions	3 deg/s
Test IV	Umbra conditions	1 deg/s

## Data Availability

The data are not publicly available due to the rules of our institute.

## Abbreviations

The following abbreviations are used in this manuscript:

ADR	Active Debris Removal
CNN	Convolutional Neural Networks
ECI	Earth Central Inertial
EPOS	European Proximity Operations Simulator
GNC	Guidance Navigation and Control
HIL	Hardware-in-the-Loop
ICP	Iterative Closest Point
LIDAR	Light Detection and Ranging
OOS	On-Orbit Servicing
PCA	Principal Component Analyses
PID	Proportional Integral Derivative
PMD	Photonic Mixer Device
RANSAC	Random Sample Consensus
SVD	Singular Value Decomposition

## Appendix A. Pitch and Yaw Error Characterization

The pitch and yaw errors plotted in Figure 7 and Figure 8 show a periodical behaviour. The data were fitted to the non linear model y(t)=Asin(ωt+ϕ)+B. The error *y* is a function of the time *t*, the amplitude *A*, the angular velocity ω, the phase ϕ and the offset *B*. The fit was done using a simplified version of the sinusfit function from this computational package: [44].

The recorded signals used to characterize the error cover less than one period for tests II and IV, and only two periods for tests I and III. Therefore, the present numerical fit is a limit case especially for the frequency estimation. The quality of the result is still enough to clearly separate the cases with a target rotating at 3 deg/s from the 1 deg/s angular velocity cases.

**Table A1:** Detailed results of sinus fit to yaw and pitch errors. Test I and II approached an illuminated the target. Test III and IV consists in approaches in darkness. The target was rotating with 3 deg/s during test I and III. The rotation velocity of the target was 1 deg/s for test II and IV. Each test was repeated five times.

Rep.	Yaw					Pitch				
	ω	A	ϕ	B	RMSE	ω	A	ϕ	B	RMSE
	[${}^{\circ }$/s]	[${}^{\circ }$]	[${}^{\circ }$]	[${}^{\circ }$]	[${}^{\circ }$]	[${}^{\circ }$/s]	[${}^{\circ }$]	[${}^{\circ }$]	[${}^{\circ }$]	[${}^{\circ }$]
Test I										
1	2.78	0.36	−180.0	2.11	0.126	2.84	0.38	0.0	−0.07	0.091
2	2.81	0.37	−180.0	2.12	0.128	2.96	0.38	−144.1	−0.07	0.077
3	2.65	0.32	−180.0	2.12	0.148	2.69	0.40	0.0	0.02	0.103
4	2.85	0.38	−180.0	2.14	0.128	2.81	0.39	−180.0	0.07	0.078
5	2.86	0.42	173.0	2.16	0.123	2.69	0.37	−180.0	0.12	0.101
Test II										
1	0.86	0.48	2.1	2.07	0.098	1.08	0.32	143.3	−0.39	0.081
2	1.09	0.33	−177.6	2.21	0.093	0.98	0.33	175.7	−0.46	0.079
3	1.05	0.31	−179.9	2.17	0.082	0.99	0.36	141.2	−0.46	0.076
4	1.02	0.27	−180.0	2.16	0.088	0.95	0.39	-173.3	−0.47	0.088
5	1.00	0.25	−178.6	2.34	0.075	0.95	0.40	177.5	−0.45	0.071
Test III										
1	2.31	0.30	−180.0	2.18	0.221	2.54	0.38	0.0	−0.37	0.125
2	2.86	0.40	0.0	2.21	0.125	2.74	0.39	0.0	−0.27	0.090
3	2.40	0.32	−180.0	2.27	0.203	2.64	0.37	−180.0	−0.23	0.104
4	2.90	0.40	0.0	2.25	0.111	2.71	0.40	−180.0	−0.17	0.086
5	2.81	0.39	-180.0	2.28	0.118	2.86	0.39	0.0	−0.23	0.071
Test IV										
1	0.99	0.18	−179.5	2.51	0.077	0.92	0.44	−71.0	−0.55	0.068
2	0.99	0.19	−179.7	2.50	0.079	0.93	0.41	−95.3	−0.57	0.068
3	0.97	0.22	−159.8	2.50	0.085	0.92	0.43	−53.0	−0.65	0.072
4	0.90	0.36	77.3	2.49	0.084	0.90	0.44	10.4	−0.63	0.109
5	0.96	0.43	−146.6	2.54	0.074	0.95	0.37	−179.2	−0.60	0.069
